# Spatiotemporal Analysis of the Coupling Relationship between Habitat Quality and Urbanization in the Lower Yellow River

**DOI:** 10.3390/ijerph20064734

**Published:** 2023-03-08

**Authors:** Jinxin Sun, Mei Han, Fanbiao Kong, Fan Wei, Xianglun Kong

**Affiliations:** 1 College of Geography and Environment, Shandong Normal University, Jinan 250358, China; 2 College of Ecology, Resources and Environment, Dezhou University, Dezhou 250323, China

**Keywords:** habitat quality, InVEST, urbanization, coupling coordination, Lower Yellow River

## Abstract

Natural habitats are damaged by human interference to varying degrees during the urbanization process, which can impede a region’s high-quality development. In this study, we examined the spatial–temporal evolution characteristics of habitat quality and urbanization in the Lower Yellow River from 2000 to 2020 using the integrated valuation of ecosystem services and tradeoffs (InVEST) model and the comprehensive indicator method. We also evaluated the coupling relationship between the habitat quality and urbanization using the coupling coordination degree model. The findings indicate the following aspects: (1) Between 2000 and 2020, the Lower Yellow River’s habitat quality was typically mediocre, with a steady declining trend. The majority of cities displayed a trend toward declining habitat quality. (2) Both the urbanization subsystem and the urbanization level in 34 cities have demonstrated a consistent growth tendency. The urbanization level is most affected by economic urbanization among the subsystems. (3) The coupling coordination degree have revealed an ongoing trend of growth. In most cities, the relationship between habitat quality and urbanization has been evolving toward coordination. The results of this study have some reference value for ameliorating the habitat quality of the Lower Yellow River and solving the coupling coordination relationship between habitat quality and urbanization.

## 1. Introduction

The ability of a habitat to supply biological communities with stable conditions is referred to as habitat quality (HQ) [1,2]. On one hand, the background circumstances of local natural resources affect the HQ. On the other hand, the HQ also depends on the intensity of outside disturbances [3]. Human intervention during the urbanization process has significantly altered natural environments. It is believed that human activity during the urbanization process directly threatens the quality of the local habitats. Severe environmental degradation has occurred in such habitats [4,5]. In an earlier study, the HQ was typically evaluated using the indicator assessment method, which built a system of indicators using data from field surveys. To measure the HQ of Chinese provinces, Fu [6] chose 12 indicators, such as soil erosion, land salinization, and solid waste contamination. However, the laborious processes of field sampling and field surveys make it challenging to apply the indicator assessment method to large-scale HQ assessments [7]. Model evaluation has increasingly grown in importance as a research method for HQ due to the maturity of technical instruments such as remote sensing systems and the quick development of HQ assessment models [8,9]. For long-term HQ monitoring, the model assessment method has a number of advantages over the integrated indicator evaluation method [10]. The habitat suitability index (HSI) model [11], social values for ecosystem services (SolVES) model [12,13], and InVEST model [14,15] are examples of common models. The InVEST model assesses biodiversity based on the quantity of the habitat exposed to external threats, which it deems to be the primary factor contributing to the degradation of HQ [16,17]. In comparison to other models, the InVEST model has better evaluation accuracy and is more convenient for data acquisition. As a result, the InVEST model is increasingly being used in dynamic habitat quality evaluations [18].

Urbanization is a concept with multiple implications. Numerous topics are covered, including populations, economies, social security, culture, and health care [19,20]. The understanding of urbanization varies among academics from various disciplines, which has a significant impact on how urbanization levels are quantified [21,22,23,24]. The comprehensive index method may quantify the urbanization level more thoroughly than other methods of evaluation. One of the most important factors in determining the urbanization level using the comprehensive indicator method is the selection of indicators. In terms of the construction of the indicator system, the study of building the urbanization indicator system from the four aspects of space, population, economy, and society has a significant impact [25]. Urbanized systems and natural ecosystems interact in a complex way. In order to systematically analyze the coupling and coordination relationship between urbanization and habitats, we chose the comprehensive indicator method to evaluate the transformation of the urbanization level in the Lower Yellow River.

The impact of urbanization on ecological environments is one of the important issues in the study of human–land relationships. Earlier studies focused more on the impacts of urbanization on single environmental factors such as air and water [26]. These environmental factors are usually closely related to human health. The natural ecosystem is a complex system which includes soil, water, organisms, and other environmental elements [27]. Since the negative impacts of intense human activities on the authenticity and stability of natural ecosystems have been widely recognized, the interaction between urbanization and natural ecosystems has gradually become the focus of scholars. In recent years, scholars have analyzed the relationship between urbanization and ecological environments from the perspectives of ecosystem services [28] and landscape fragmentation [29,30,31], but the research on the coupling relationship between HQ and urbanization is insufficient. The quality of a habitat is determined by the natural background condition of habitat and the intensity of external threats represented by human activities. The quantitative study of the coupling relationship between HQ and urbanization plays an important role in coordinating the human–land relationship and is worthy of further study.

The Yellow River Basin is an important biological barrier in China, and it is vital to coordinate basin protection and development issues in order to ensure long-term national stability [32]. The ecological preservation and sustainable development of the Yellow River Basin have recently emerged as crucial national strategies. The Yellow River Basin’s ecological conservation is a hot topic of research right now [33]. Studies on the relationship between the ecological environment and urbanization in the Yellow River Basin have primarily concentrated on the basin as a whole or on arid and semi-arid regions such as Shaanxi and Ningxia [34,35,36]. There haven’t been many studies on the relationship between HQ and urbanization in the Lower Yellow River. The Lower Yellow River has higher resident population density and gross regional product in the Yellow River Basin. The Lower Yellow River has become more essential for its ability to sustain regional economic growth and habitat protection [37]. The interactive coupling link between HQ and urbanization development in the Lower Yellow River must be quantitatively studied. Understanding the characteristics of the coupling coordination involving HQ and urbanization is strategically important for promoting HQ and high-quality economic growth in the Lower Yellow River. This is also a key issue for coordinating the interactions between people and the land and fostering sustainable development.

Based on this, the two primary scientific questions addressed in this study are as follows: (1) How did the coupling relationship between the natural habitat and urbanization evolve in the Lower Yellow River from 2000 to 2020? (2) How do the coupling relationships between different urbanization subsystems and the ecological environment differ? In order to tackle these scientific questions, our study uses the InVEST model to evaluate the HQ of the Lower Yellow River from 2000 to 2020, generates urbanization indicators to describe the regional urbanization level using socioeconomic data, and uses the coupling and coordination degree model (CCDM) to reveal the relationship between HQ and the urbanization level. More particularly, our research aims to (1) describe the spatial and temporal distribution of the habitat quality in the Lower Yellow River from 2000 to 2020; (2) assess the urbanization level of urban agglomerations in the Lower Yellow River from population, economics, social security, and space perspectives; and (3) disclose the spatiotemporal evolution of the coupling coordination relationship between HQ and urbanization in the Lower Yellow River.

## 2. Data Material Sources and Research Methods

### 2.1. Definition of the Study Area

**Figure 1:**
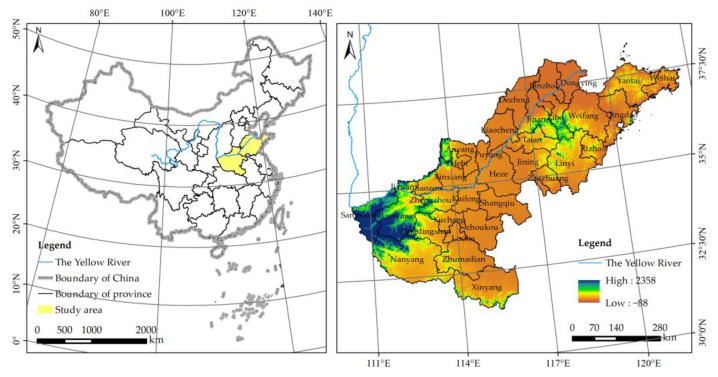
Location of the study area.

Geographically speaking, the Lower Yellow River refers to the stretch of the river that runs through the provinces of Shandong and Henan from Taohuayu to the estuary. Meanwhile, the integrity of the administrative unit should be preserved as much as is feasible in the coupling research of the ecological environment and urbanization. In order to do this, we adhered to the maxim of “taking the natural watershed of the Yellow River as the core scope and protecting the integrity of the administrative unit in every feasible way” [38]. We defined the Lower Yellow River’s geographic scope as 34 cities in the provinces of Henan and Shandong (Figure 1).

### 2.2. Data Sources and Processing

The five epochs of land use data of the Lower Yellow River were obtained from the Resource and Environment Science and Data Center of the Chinese Academy of Sciences (https://www.resdc.cn/), accessed on 1 September 2022. With the exception of marshes, we combined the six secondary land use classifications as unused land (the primary land use classification) based on the original categorization system. The socioeconomic data needed for the construction of the urbanization index system were obtained from the Shandong Province Statistical Yearbook, the Henan Province Statistical Yearbook, and the China Urban Statistical Yearbook.

### 2.3. Methods

#### 2.3.1. Evaluation of Habitat Quality

We calculated the habitat quality (HQ) index based on the InVEST model. The HQ is based on the degree to which each land use type is compatible with the habitat, manner, and radius of the threat source’s influence and the susceptibility of the land class to the danger source [39]. The formula is shown below as follows:

(1) $$Q_{xj}=H_{j}\left[1-\left(\frac{D_{xj}}{D_{xj}+k}\right)\right]$$

where *Q_xj_* represents the habitat quality index of raster cell *x* of land use type *j*, and *H_j_* is the habitat suitability of land use type *j*. Here, *D_xj_* is the habitat degradation index of raster cell *x* of land use type *j*, and it is calculated using Equation (2); *k* is a semi-saturated parameter whose value is equal to half of the maximum value of the habitat degradation index:

(2) $$D_{xj}=\sum_{r=1}^{R}\sum_{y=1}^{Yr}\left(\frac{w_{r}}{\sum_{r=1}^{R}w_{r}}\right)r_{y}i_{rxy}\beta_{x}S_{jr}$$

where *R* is the number of threat sources, *y* represents a grid cell in the threat source layer *r*, *Y_r_* is the total number of grid cells of the threat source *r*, *w_r_* is the weight of threat source *r*, *S_jr_* is the sensitivity of land use type *j* to threat source *r*, and *β_x_* is the degree of legal protection. Here, *r_y_* is an auxiliary value used to determine the position of the threat source raster in the layer. In the threat source layer, if the raster *y* belongs to the threat source, *r_y_* = 1, otherwise *r_y_* = 0. As shown in the equation below, *i_rxy_* is calculated in two ways, as a linear decline and exponential decline:

(3) $$i_{rxy}=1-\left(\frac{d_{xy}}{d_{r\max}}\right)\;\mathrm{if}\;\mathrm{linear}$$

(4) $$i_{rxy}=\exp\left(-2.99\left(\frac{d_{xy}}{d_{r\max}}\right)\right)\;\mathrm{if}\;\mathrm{exponential}$$

where *d_xy_* represents the distance between raster *x* and *y*; *d_r_*_max_ represents the maximum influence distance of threat source *r*.

From the above equations, we can conclude that identifying the threat sources and threat parameters is crucial for the efficient operation of the InVEST model. The Lower Yellow River is an important grain-producing area in China. This region includes the urban agglomerations of Zhongyuan and the Shandong peninsula. In this situation, the major threats to the environment are cropped land and construction land because they are directly tied to human activities. The unutilized land’s natural background state is poor, posing a danger to the surrounding habitat. Given the features of the study area, relevant studies, and experts’ opinions, we chose paddy field, dry land, urban construction land, rural residential land, and other land use types as threat sources. Table 1 and Table 2 show the model input parameters, whose values are derived from the InVEST model guidebook, scholars’ research [40,41,42], and experts’ opinions.

#### 2.3.2. Evaluation of Urbanization

**Figure 2:**
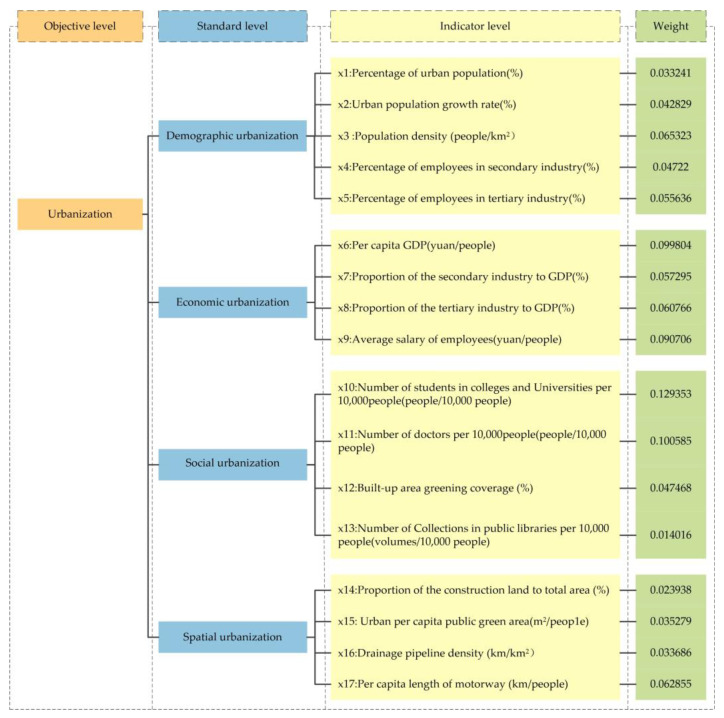
The Lower Yellow River’s urbanization evaluation index system.

Considering the data accessibility and comparability, we selected 17 indicators from the population, economic, social security, and space categories. The urbanization evaluation index system is shown in Figure 2. We utilized the linear weighted sum approach to evaluate the urbanization levels. The calculation formula is shown below:

(5) $$U_{i}=\sum_{j=1}^{n}w_{j}\times U_{ij}$$

Here, *w_j_* stands for the indicator *j*’s weight, and *U_ij_* for the indicator *j*’s normalized value in city *i*. Each indicator’s weights were calculated using the entropy weight approach.

#### 2.3.3. Coupling Coordination Degree Model

**Figure 3:**
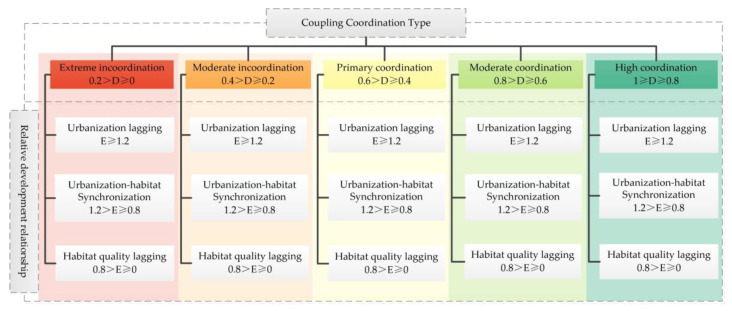
Coupling coordination types of urbanization and HQ.

The coupling coordination degree model, which contains the coupling degree *C*, the comprehensive evaluation index *T*, and the coupling coordination degree *D*, is able to quantify the degree of coherence in the system’s development [43]. However, it is difficult to objectively reflect the level of synergy between systems by relying on the coupling degree alone, so *T* and *D* are defined to reflect the degree of system contribution to coordination [44]. In order to analyze the status of HQ and urbanization in the coupling coordination link, a synchronous development index *E* is constructed to explain the synchronous or lagging state of the two processes:

(6) $$C=2\sqrt{\frac{U_{1}U_{2}}{{\left(U_{1}+U_{2}\right)}^{2}}}$$

(7) $$T=aU_{1}+bU_{2}$$

(8) $$D=\sqrt{CT}$$

(9) $$E=U_{1}/U_{2}$$

where *U*_1_ and *U*_2_ denote the HQ and urbanization level, respectively; *a* and *b* are coefficients to be determined; *a* + *b* = 1, *a*, *b* are used to characterize the importance of the HQ and urbanization level. Referring to the studies by Ma et al. [45] and Tang et al. [46], we considered the habitat quality system as equally important as the urbanization system, so we set *a*, *b* = 0.5. The values of *C*, *D* are between 0 and 1. The type of coupling coordination was divided as shown in Figure 3.

## 3. Results

### 3.1. Variations in Habitat Quality through Time and Space along the Lower Yellow River

**Figure 4:**
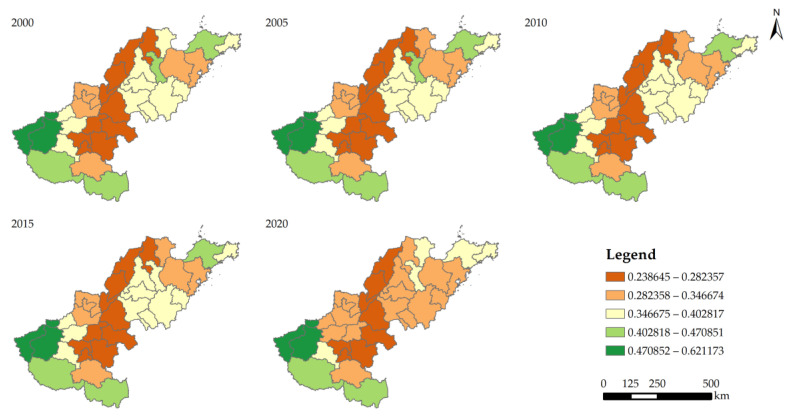
Habitat quality of each city in the Lower Yellow River from 2000 to 2020.

Using the InVEST model, we evaluated the Lower Yellow River’s HQ status in the years 2000, 2005, 2010, 2015, and 2020. We divided the HQ into five categories: low (0.0–0.02), relatively low (0.2–0.4), medium (0.4–0.6), relatively high (0.6–0.8), and high (0.8–1). The findings indicated that the Lower Yellow River’s HQ was generally in poor condition and has been declining steadily from 2000 to 2020, with mean values of 0.368, 0.366, 0.365, 0.364, and 0.357, respectively. The HQ values of several cities in the research area varied dramatically between 2000 and 2020, ranging from 0.239 to 0.621. Sanmenxia consistently maintained the highest level of HQ, followed by Luoyang and Jiyuan at various points in time. The HQ values of Nanyang, Xinyang, Yantai, Pingdingshan, and Zibo were all higher than the study area average, while more than 67% of the cities were lower to varying degrees (Figure 4).

**Figure 5:**
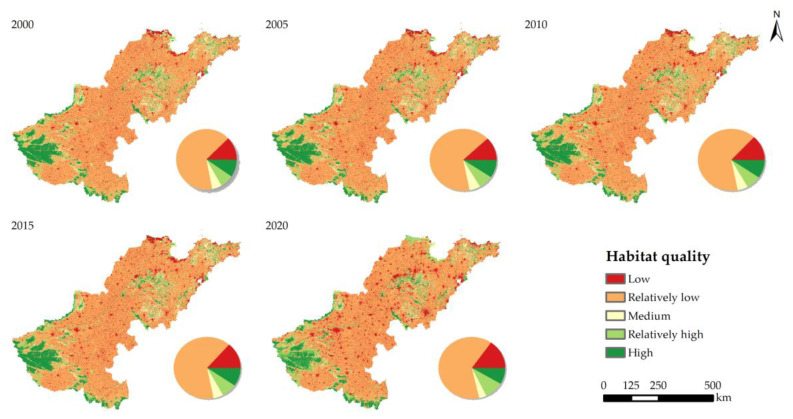
Spatial distribution of habitat quality along the Lower Yellow River from 2000 to 2020.

In terms of the spatial distribution, the HQ in the study region had a pattern of poor HQ in the center and high HQ around it (Figure 5). The research region’s HQ grades are primarily rather low, with more than 60% of the land falling into this category, which is mostly spread in the low-altitude plains. About 13% of the region is low-grade, which includes construction land and some cultivated land that is scattered. Approximately 17% of the areas are of relatively high and high grades; these areas primarily include wetlands near rivers, lakes, and seashores, as well as forest and grassland areas at higher altitudes.

Regarding temporal variations in HQ, more than 19% of the regions displayed a decline in the HQ grade, almost 15% exhibited an increase in the HQ grade, and roughly 66% were unaltered. The Lower Yellow River showed a significant change in HQ between 2000 and 2020, which was mostly reflected in the trend of the cities with poorer HQ. The number of cities with reduced HQ increased from 27 to 33 and subsequently declined to 24 during the study period. The HQ of Kaifeng, Zhumadian, Xinyang, Binzhou, Dongying, Weifang, Jiyuan, and Nanyang improved with time, while the HQ of the other cities in the research area declined to varying degrees.

### 3.2. Variations in Urbanization through Time and Space along the Lower Yellow River

**Figure 6:**
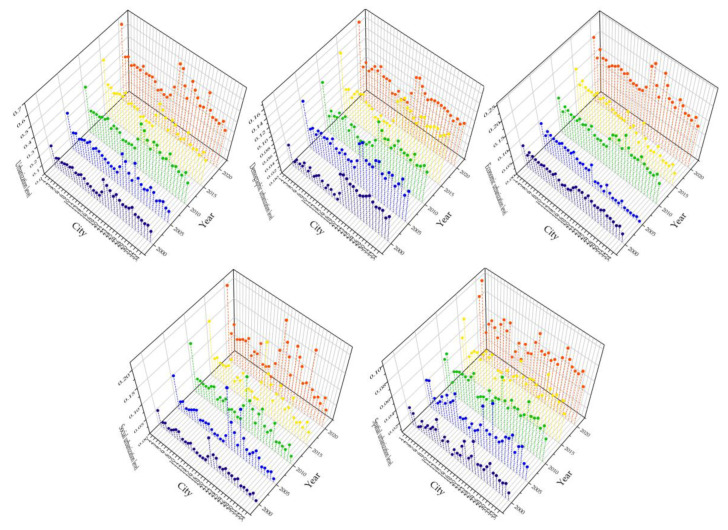
Urbanization levels and subsystem urbanization levels of each city in the Lower Yellow River from 2000 to 2020: (1) Zhengzhou; (2) Kaifeng; (3) Luoyang; (4) Pingdingshan; (5) Anyang; (6) Hebi; (7) Xinxiang; (8) Jiaozuo; (9) Puyang; (10) Xuchang; (11) Luohe; (12) Sanmenxia; (13) Nanyang; (14) Shangqiu; (15) Xinyang; (16) Zhoukou; (17) Zhumadian; (18) Jiyuan; (19) Jinan; (20) Qingdao; (21) Zibo; (22) Zaozhuang; (23) Dongying; (24) Yantai; (25) Weifang; (26) Jining; (27) Taian; (28) Weihai; (29) Rizhao; (30) Linyi; (31) Dezhou; (32) Liaocheng; (33) Binzhou; (34) Heze.

Between 2000 and 2020, the urbanization levels of 34 cities in the Lower Yellow River progressively grew, as did regional disparities in the urbanization levels between cities. During the study period, the urbanization levels of Xinyang, Zhumadian, Zhouko, Nanyang, Shangqiu, and Heze were consistently lower than the study area’s average, while those of Jinan, Qingdao, Zhengzhou, Weihai, Yantai, and Dongying were consistently higher than the average (Figure 6). The urbanization levels in the Lower Yellow River region ranged from 0.084 to 0.307 between 2000 and 2005, and the urbanization growth was rather sluggish. The urbanization levels of Jinan, Qingdao, Weihai, and other cities dramatically grew between 2005 and 2010, widening the disparity in regional urbanization levels. The overall urbanization level of the Lower Yellow River significantly increased from 2010 to 2020, spurred by Jinan, Qingdao, Zhengzhou, and other cities, and the regional urbanization has evolved quickly, with an urbanization level between 0.312 and 0.636.

The examination of the four urbanization subsystems revealed that although each subsystem’s urbanization level in the Lower Yellow River varied considerably, they all displayed a consistent upward tendency (Figure 6). The level of economic urbanization, which is substantially larger than that of the other three subsystems, is the factor that most affects the amount of urbanization along the Lower Yellow River. In comparison to other cities in the research area, Jinan, Qingdao, Yantai, Dongying, Zhengzhou, and Luoyang have obviously higher degrees of economic urbanization, whereas Liaocheng, Heze, Dezhou, and Linyi have lower levels—falling below the regional average in all periods.

In terms of social urbanization, this is the fastest growing and most regionally diversified subsystem. The social urbanization is more advanced in Jinan, Zhengzhou, and Weihai, followed by Jinan, Weihai, and Zhengzhou, but it is consistently less advanced in Zhoukou, Zhumadia, Nanyang, and Heze. The spatial urbanization increased more quickly between 2000 and 2010 and less rapidly between 2010 and 2020. Higher spatial urbanization levels can be seen in Qingdao, Weihai, Kaifeng, and Zhengzou, followed by Rizhao, Binzhou, and Luohe, while Sanmenxia, Xinyang, and Nanyang have lower levels. In comparison to the other subsystems, the increase in demographic urbanization was quite small. The rate of demographic urbanization rose by only 13% between 2000 and 2020, whereas the rate of urbanization for the other three subsystems was more than twice as high in 2020 as it was in 2000. Zhoukou, Shangqiu and Heze have comparatively low levels of demographic urbanization, whereas Zhengzhou, Jinan, and Weihai have quite high levels.

### 3.3. Coupling Coordination Relationship between Urbanization and Habitat Quality in the Lower Yellow River

**Figure 7:**
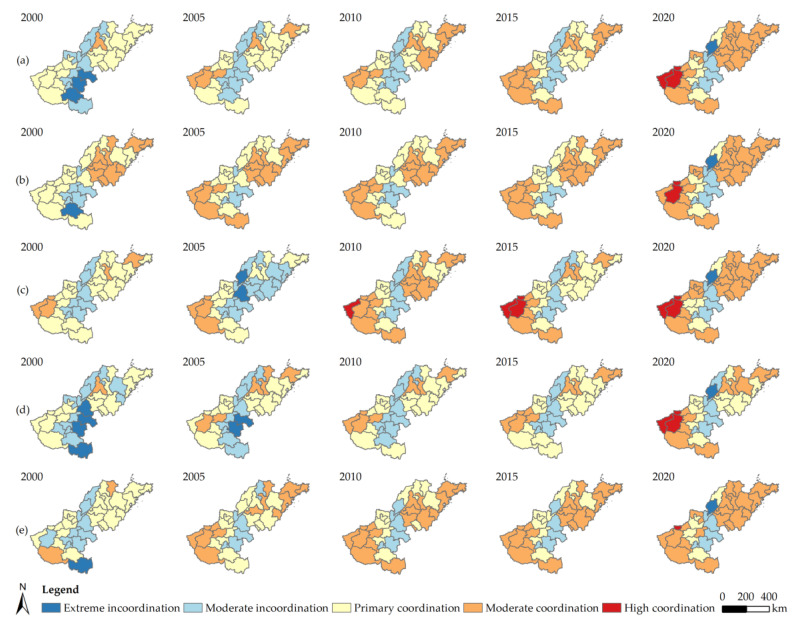
Coupling and coordination types of urbanization levels and subsystem urbanization levels with HQ for each city in the Lower Yellow River from 2000 to 2020: (**a**) urbanization level; (**b**) demographic urbanization level; (**c**) economic urbanization level; (**d**) social urbanization level; (**e**) spatial urbanization level.

The coupling coordination degree (CCD) between the HQ and urbanization level exhibited a consistent upward trend from 2000 to 2020, and the variation in CCD values between cities was striking. Only a few cities showed a decline in the coupling coordination relationship involving HQ and urbanization according to Figure 7, which shows that the majority of cities are moving in a coordinated manner. The vast majority of cities had a CCD below 0.6 in 2000, which is a relatively low value. Moderate coordination and high coordination levels were not included in the coupling coordination category. There were 17 cities with primary coordination, 14 with moderate incoordination, and 3 with extreme incoordination. The number of cities with extreme incoordination decreased to zero between 2000 and 2005, whereas the number of cities with moderate incoordination decreased and cities with moderate coordination increased from zero to seven. From 2005 to 2015, the number of cities with moderate incoordination and primary coordination dropped. The number of cities with moderate coordination increased. The level of CCD in the study area further improved between 2015 and 2020, whereas it declined in some cities. The number of cities with primary coordination and moderate incoordination declined, and the number of cities with high coordination increased from 0 to 3. However, the degree of coupling coordination in Liaocheng decreased from moderate incoordination to extreme incoordination.

In terms of the CCD between the HQ and the four subsystems of urbanization, the CCD of the cities in the Lower Yellow River typically displayed a rising tendency. In the coupling coordination relationship between the four urbanization subsystems and HQ, the social urbanization CCD increased the fastest, the CCD values of the economic and demographic urbanization were relatively high, and the CCD of the spatial urbanization exhibited the least regional variation. The coupling and coordination interactions between various urbanization subsystems and HQ differ significantly, yet in the same city, the relationships between the four urbanization subsystems and HQ are largely positive. In a city, if the CCD of the demographic urbanization level with HQ is relatively high, the other three urbanization subsystems with HQ are also likely to have a high CCD. There were four cities, however, where the CCD between the urbanization subsystems and HQ did not follow the above pattern. For the demographic urbanization and spatial urbanization, Luoyang had a low CCD, whereas the economic urbanization and social urbanization had a high CCD. Dezhou, Binzhou, and Heze had a relatively high CCD for demographic urbanization but a low CCD for other urbanization subsystems. Among the remaining cities, Zhengzhou, Sanmenxia, Jinan, and Weihai all had extraordinarily high CCD values for subsystem urbanizations, whereas Luohe, Shangqiu, Zhoukou, and Liaocheng all had relatively low CCD values for subsystem urbanizations.

**Figure 8:**
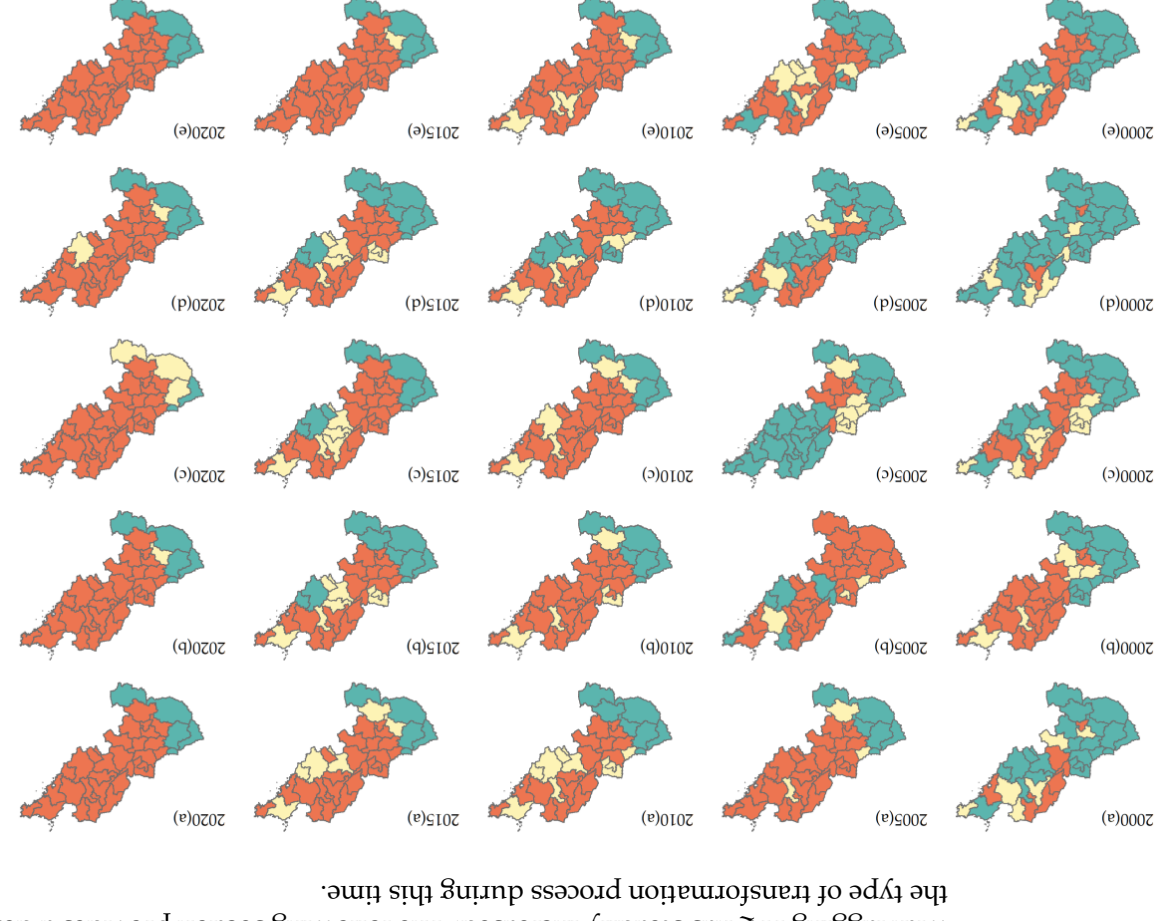
Relative development relationships of the urbanization level and subsystem urbanization level with HQ for each city along the Lower Yellow River between 2000 and 2020: (**a**) urbanization level; (**b**) demographic urbanization level; (**c**) economic urbanization level; (**d**) social urbanization level; (**e**) spatial urbanization level.

Using Equation (7), we divided the coupling coordination characteristics between HQ and urbanization in the Lower Yellow River into three categories: urbanization lagging, urbanization–HQ synchronization, and HQ lagging. From 2000 to 2020, Luohe, Puyang, Liaocheng, Heze, Dezhou, and Binzhou all belonged to HQ lagging, whereas Sanmenxia, Nanyang, Xinyang, Luoyang, and Jiyuan all belonged to urbanization lagging. The abundance of resources in these cities has a significant impact on this. Figure 8 shows that the number of cities with lagging urbanization has steadily declined, while the number of cities with lagging HQ has steadily increased. The following section provides a description of the type of transformation process during this time.

In 2000, there were 24 cities in the research region that belonged to the urbanization lagging category, accounting for more than 70% of all cities. The environment received little effect from anthropogenic activities throughout this time, and the habitat’s condition was fairly positive. The Lower Yellow River’s urbanization level is relatively low, and improving inhabitants’ living conditions and stimulating quick economic growth remain the primary goals of urbanization development. Between 2000 and 2005, 50% of all cities underwent a type shift, primarily moving from urbanization–HQ synchronization and urbanization lagging to HQ lagging. From 2005 to 2010, a transition from urbanization lagging to urbanization–HQ synchronization mostly occurred, with around 33% of the total number of cities experiencing this shift. During this timeframe, the study area’s level of urbanization continuously increased, the gap between urbanization and HQ gradually shrank, and the number of cities with urbanization–HQ synchronization greatly increased. However, as the urbanization levels rose quickly, a possible risk of urban growth affecting HQ slowly became apparent. Between 2010 and 2020, the majority of cities underwent a type change, changing from urbanization–HQ synchronization to HQ lagging. Over the course of the study period, fewer cities experienced a type shift, and the coupling coordination relationship between urbanization and HQ gradually stabilized.

## 4. Discussion

In this study, the mean value of the HQ in the Lower Yellow River fell from 0.368 to 0.357 between 2000 and 2020, with a definite downward trend. Among the grades of HQ in the Lower Yellow River, the low-grade areas increased, while the high-grade areas decreased. This may be attributed to the increase in the area of threatening sources represented by building land and the decrease in the area of land use types with higher ecological value, which is consistent with the conclusions found by Zhou et al. [47] and Lin et al. [48]. Therefore, in order to promote the long-term optimization of the ecological environment, governments and pertinent departments at all levels should adhere to the principles of environment-first and eco-friendly development, judiciously manage the growth of built-up areas, and stop the inefficient and unequal distribution of construction land from eroding ecological land [49].

It is worth noting that the overall habitat quality in the Lower Yellow River showed a downward trend, but the area changes in the regions of a relatively high grade and medium grade did not follow this rule. Compared with the area changes of other grades, the relatively high-grade area increased significantly while the medium-grade area decreased significantly from 2015 to 2020. From the change in quantity of each grade, the increase in relatively high-grade areas came mainly from medium-grade areas. This shift may be related to environmental governance in areas of medium grade. This phenomenon shows that ecological restoration in areas of medium habitat quality is one of the most effective ways to improve the overall habitat quality in the region. In order to achieve the goal of improving habitat quality, we should vigorously promote the “urban and rural greening” action and focus on environmental governance in areas with moderate habitat quality [50]. In such areas, the government should promote the development of regional habitat quality in a positive direction by expanding public green spaces, creating urban green parks, and buffering shelterbelts.

In terms of the CCD between HQ and urbanization, cities in the middle of the Lower Yellow River usually fall into the moderate incoordination and extreme incoordination categories. Their level of urbanization is low. Additionally, these cities’ HQ is mediocre in comparison to their urbanization. Cities with lagging HQ, such as those in the center Lower Yellow River, should identify the unsolved habitat issues as soon as possible and rectify the negative impacts of urbanization on the ecosystem. Next, they should address the subjective and objective problems that prevent high-quality urbanization and provide favorable socioeconomic conditions for habitat improvements. The remaining cities essentially fall into two categories, moderate coordination and high coordination, with minimal variance in terms of urbanization and HQ. These cities are further classified as having lagging urbanization and lagging HQ based on the relative states of the two systems. Cities that are part of the HQ lagging category should be aware of any potential HQ problems brought on by urbanization. For sustainable urban development, the government must provide an eco-friendly platform [51,52]. In light of their unique circumstances, cities with different relative development relationships between HQ and urbanization should create their urban development strategies.

In terms of the coupling relationship between the four subsystems and HQ, the CCD of the economic urbanization and demographic urbanization is higher. Yun [53] evaluated the coupling coordination between the ecological environment and urbanization in the Yellow River Basin and found that the coupling coordination index of the demographic urbanization was lower than that of the other three urbanization subsystems. Yun’s conclusion is somewhat different from the results of this study, where we highlighted the evolution of the coupling coordination relationship between HQ and urbanization in regions with high levels of demographic and economic urbanization. The coupling coordination relationship between the demographic subsystem and HQ in the Lower Yellow River Basin is very different from that in the Yellow River Basin. This important difference also indicates that the study of the coupling relationship between HQ and urbanization in the Lower Yellow River is of great significance for revealing the coupling relationship between urbanization and HQ in densely populated and economically developed areas.

This study looked at the spatial and temporal evolution of HQ and urbanization and their interactions, which can operate as a factual support for the high-quality development of socioeconomic activities and the environment in the Lower Yellow River. Additionally, this study could serve as a reliable source for determining metrics for HQ evaluations and for investigating the connections between HQ and urbanization in comparable places. However, our study still has several limitations. To begin with, the range of indicators available is limited due to the accuracy of publicly available urbanization-related data. In further research, we will try to obtain more indicators to enrich the urbanization indicator system. Second, the InVEST model is one of the most critical tools for habitat quality assessments and has been broadly applied in large-scale and meso-scale habitat quality studies. However, the InVEST model still has the limitations, in that it ignores the habitat quality variation among the same habitats and cannot fully reflect the actual habitat quality conditions. Finally, our study did not include the driving factors. Along with our upcoming research, we will attempt to delve deeper into the drivers of the relationship between HQ and urbanization in the Lower Yellow River.

## 5. Conclusions

We first characterized the spatial and temporal variations in HQ and urbanization levels using the InVEST model and the comprehensive indicator method, and then we applied the CCDM to tackle their interaction. From 2000 to 2020, the studies revealed an overall poor state of HQ along the Lower Yellow River, with a continuous downward tendency. More than 60% of the study areas had a low HQ grade. The pattern of HQ in the Lower Yellow River was poor in the middle and high around the edges. Significant disparities existed in the HQ of various cities, and while most cities displayed a trend toward declining HQ, the number of cities that did so gradually declined.

The 34 cities’ urbanization levels, as well as the urbanization levels of each subsystem, showed a stable growth tendency in terms of temporal variability in urbanization levels. Between 2000 and 2020, Jinan, Qingdao, and Zhengzhou showed higher urbanization levels, while Xinyang, Nanyang, and Heze showed lower urbanization levels. Economic urbanization has the greatest impact on the Lower Yellow River’s urbanization level, while social urbanization is the fastest expanding subsystem with the greatest regional disparity.

The CCD of the Lower Yellow River showed a consistent upward tendency between 2000 to 2020. In most cities, the relationship between HQ and urbanization moved in the direction of coordination, while only a few cities saw a decline in the coupling coordination relationship. The spatial distribution of the CCD is characterized by high values in the east and west and low values in the middle, meaning that the eastern part of Henan Province and the western part of Shandong Province have high CCD values, while the junction of these two provinces has low values of CCD. The number of cities with moderate coordination has increased significantly, while the number of cities with moderate incoordination and extreme incoordination has decreased. The number of cities with lagging urbanization has decreased, while the number of cities with lagging HQ has increased. On the whole, the evolution of the coupling coordination relationship between HQ and urbanization is positive. However, the HQ in the Lower Yellow River has gradually lagged behind urbanization, which is a potential threat to the coupling coordination between regional HQ and urbanization. This phenomenon deserves close attention. The coupling coordination levels between the four urbanization subsystems and HQ differ significantly. The CCD levels of demographic and economic urbanization are particularly high among the subsystems. Our research is critical for improving the habitat quality and coordinating the relationship between the habitat quality and urbanization in the Lower Yellow River, and it can serve as a scientific reference for government agencies’ ecological regulations.

## Figures and Tables

**Table 1:** Maximum impact distances, weights, and spatial recession types of threat sources.

Threat Source (*r*)	Maximum Threat Distance (*d_r_*_max_)/km	Weight (*w_r_*)	Decay
Paddy field	1	0.5	linear
Dry land	1	0.5	linear
Urban construction land	8	1	exponential
Rural residential land	4	0.7	exponential
Other construction land	9	0.9	exponential
Other unutilized land	1	0.3	linear

**Table 2:** Habitat suitability of land use types and their sensitivity to threat sources.

Land Type (*j*)	Habitat Suitability (*H_j_*)	Sensitivity (*S_jr_*)					
		Paddy Field	Dry Land	Urban Construction Land	Rural Residential Land	Other Construction Land	Other Unutilized Land
Paddy field	0.4	0	0.7	0.3	0	0	0.7
Dry land	0.3	0	0.6	0.2	0	0	0.6
Forest land	0.9	0.6	0.9	0.5	0.6	0.6	0.85
Scrub woodland	0.8	0.5	0.85	0.45	0.5	0.5	0.75
Sparse woodland	0.75	0.5	0.85	0.45	0.5	0.5	0.75
Other woodland	0.65	0.45	0.85	0.4	0.45	0.45	0.7
High-coverage grassland	0.7	0.55	0.9	0.5	0.55	0.55	0.85
Medium-coverage grassland	0.6	0.5	0.85	0.45	0.5	0.5	0.75
Low-coverage grassland	0.55	0.45	0.8	0.4	0.45	0.45	0.75
River	0.9	0.6	0.9	0.5	0.6	0.6	0.85
Lake	1	0.6	0.9	0.5	0.6	0.6	0.85
Reservoir	0.7	0.55	0.8	0.45	0.55	0.55	0.75
Mudflat	0.5	0.4	0.75	0.4	0.4	0.4	0.7
Beach	0.5	0.4	0.75	0.4	0.4	0.4	0.7
Swamp	0.55	0.45	0.8	0.4	0.45	0.45	0.7
Sea	0.85	0.6	0.6	0.85	0.7	0.9	0.5
Other unutilized land	0.15	0.35	0.35	0.55	0.4	0.55	0

## Data Availability

The data presented in this research are accessible from the corresponding author upon request. Since some of the data are being used in other, as of yet unpublished investigations, it is not possible for the general public to access them.
